# Gait Pattern Differences Among Children With Bilateral Cerebral Palsy

**DOI:** 10.3389/fneur.2019.00183

**Published:** 2019-03-12

**Authors:** Małgorzata Domagalska–Szopa, Andrzej Szopa

**Affiliations:** ^1^Department of Medical Rehabilitation, School of Health Sciences in Katowice, Medical University of Silesia, Katowice, Poland; ^2^Department of Physiotherapy, School of Health Sciences in Katowice, Medical University of Silesia, Katowice, Poland; ^3^Medical Rehabilitation Center Neuromed SC, Katowice, Poland

**Keywords:** postural patterns, Gillette Gait Index, cluster analysis, cerebral palsy, gait patterns

## Abstract

**Background:** The positive findings from our previous studies, which revealed the link between postural and gait patterns in children with unilateral cerebral palsy (CP) were very encouraging for recognition this relationship in children with bilateral cerebral palsy (CP). Therefore, the objective of this study was to evaluate whether different gait patterns corresponding to postural patterns in children with bilateral CP could be statistically significant according to a cluster analysis.

**Methods:** Fifty-eight participants with bilateral CP and 45 matched children with typical growth and development. The participants walked barefoot along a treadmill at their own pace. Three-dimensional kinematic data were collected using the Measuring System for Motion Analysis. To characterize gait patterns, the Gillette Gait Index (GGI) and its 16 distinct gait parameters were used. The participants were divided into four subgroups according to their postural patterns.

**Results:** A cluster analysis revealed 4 gait patterns corresponding to postural patterns: (1) normal gait pattern corresponded to neutral posture; (2) balanced gait pattern corresponded to balanced posture; (3) lordotic gait pattern corresponded to lordotic postural pattern; (4) swayback gait pattern corresponded to backward-leaning posture. There were significant differences in mean GGI and various clusters in the 8 GGI gait parameters: cadence, mean pelvic tilt; mean pelvic rotation, minimum hip flexion, peak hip abduction in swing; knee flexion at initial contact, and peak dorsiflexion in stance.

**Conclusion:** Our results showed that gait discrepancies among children with bilateral CP were not simply a result of lower limb kinematic deviations in the sagittal plane. Information on different gait patterns could improve early therapy in children with bilateral CP before abnormal gait patterns are fully established.

## Introduction

Cerebral palsy (CP) comprises a group of permanent disorders of movement and postural development that cause activity limitations. These changes are attributed to non-progressive disturbances of the developing fetal or infant brain (1). However, abnormal gross and fine motor disorders including disturbances of sensation, perception, cognition, communication, and behavior are core features of CP. Additionally, epilepsy and secondary musculoskeletal problems often accompany CP. Motor impairments in CP manifest as spasticity, dystonia, muscle contractures, bony deformities, coordination problems, loss of selective motor control, and muscle weakness (2). The cause of these impairments is multifactorial.

CP can be unilateral or bilateral. Unilateral CP (UCP) is a sub-type affecting the limbs on one side of the body, whereas bilateral CP (BCP) affects the limbs on both sides of the body. Although this classification includes only the extremities and is based on subjective comparisons of severity in the arms and legs, SCPE recommends that all body regions (including the trunk and pelvis) should be specified in terms of posture and movement impairments (3).

One of the most striking features observed in children with CP is the diversity of postural and gait deviations. Even children with UCP, who appear to be a relatively homogeneous group in terms of body posture, can be distinguished by different postural and gait patterns (4–6). Our previous studies also indicated a strong correlation between postural and gait patterns in children with UCP (7, 8). Nevertheless, posture among children with BCP is much more varied than among children with UCP. Our previous study revealed that their postural patterns can be defined. Based on sagittal posture misalignments, three types of postural patterns are recognized in children with BCP: (1) lordotic postural pattern corresponding to forward-leaning posture; (2) swayback postural pattern corresponding to backward-leaning posture; and (3) balanced postural pattern corresponding to balanced posture (9).

Although the posture and gait patterns in BCP differ vastly among patients, several characteristic patterns are observed. Numerous classification schemes were proposed in the literature to describe common gait deviations in BCP (10–17). The classification most commonly described in the international literature is that by Rodda and Graham based on sagittal plane kinematics considering the ankle, knee, hip, and pelvis. Four gait patterns are recognized in BCP: true equinus, jump gait, apparent equinus, and crouch gait. Most classifications systems, including the aforementioned, classify gait patterns based on deviations in sagittal plane lower limb gait kinematics without accounting for pelvic kinematics and truncal posture (13, 14).

Although quantitative 3DGA is an excellent indicator of gait dysfunction in children with CP, the 3DGA produces a large amount of data. To overcome this problem, multivariate gait indices for quantifying deviations from normal gait have been introduced. One of the most commonly used indexes for quantifying deviations from normal gait in children with CP is the GGI. GGI, formerly called the Normalcy Index (18), is a summary measure that accounts for 16 clinically important gait parameters (including temporal, spatial, and kinematic parameters). GGI values are reportedly <46 in children without disabilities, and range from 32 to 1,827 in children with CP, including children with BCP, who range from 28.46 to 1322.3 (18, 19). Higher GGI values indicate greater deviation from the gait of able-bodied individuals. More severe diagnoses, on average, would correspond to more gait abnormalities and, thus result in higher index scores (18–21).

Our previous study revealed different postural and gait patterns in children with UCP and documented the dependence of gait patterns on body posture. According to SCPE recommendations and the promising findings of our previous research, we hoped to identify clinical clusters helpful for anticipating some rehabilitative interventions.

The objective of this study was to evaluate whether different gait patterns corresponding to postural patterns in BCP were statistically significant in a cluster analysis. We aimed to estimate degrees of deviation from normal gait within the gait patterns in BCP. We used the GGI to determine the differences in gait biomechanics (spatio-temporal and kinematics).

## Methods

### Participants

The study was approved by the Bioethics Committee of our institution. The children were subjected to examinations after obtaining parental informed written consent. All work was performed in accordance with the Code of Ethics of the World Medical Association (Declaration of Helsinki).

We recruited 60 ambulatory children with BCP who were patients of local pediatric rehabilitation centers. All participants met the following inclusion criteria: (1) diagnosis of BCP; (2) age >7 years (to minimize the incidence of kinematic parameter instability); (3) ability to walk without assistive devices and orthoses; (4) ability to follow verbal directions. The exclusion criteria were: (1) spasticity management using Botulinum Toxin A (BtxA) injection 6 months prior to evaluation; (2) lower extremity surgical procedures, (3) hip dislocation; (4) currently pharmacological treatment with oral antispasticity drugs; and (5) uncontrolled seizures.

Two participants were excluded (refused to undress for the examination) so the final study group consisted of 58 participants (25 girls and 33 boys) aged 7–13 years (mean age = 10.6 y; SD 2 y). Participants were classified into Gross Motor Function Classification System (GMFCS) level I (*n* = 34) and II (*n* = 24).

An age- and sex-matched sample of 45 children with typical development and no known history of neurological or orthopedic diseases (18 girls and 27 boys; mean age 10.2 y; SD 2 y) were included.

### Testing Procedure

Three-dimensional (3D) kinematic data were collected using the Compact Measuring System for 3D Real-Time Motion Analysis system (CMS-HS 3D, Zebris Medizintechnik GmbH, Germany) based on 15 active ultrasonic markers (5 triplicate ultrasound markers) using WinGait software (7, 8). Gait data were recorded as the participants walked on a designated treadmill (Alfa XL, Kettler, Germany).

Before the gait analysis, the following anatomical landmarks were identified: hip joint center; knee center (medial and lateral femoral epicondyle); ankle rotation center (internal and external); forefoot landmark (between the second and third metatarsals); and rear foot (heel). Before data collection, all participants were instructed to walk barefoot on the treadmill at their own pace for 5 min. The speed of the treadmill was adjusted to each child's ability and natural gait. Typical overground walking speed (spontaneous) of and time taken to walk 10 m by each subject were collected before the gait analysis. Based on the spontaneous speed of walking, treadmill belt speeds were calculated as values in kilometers per hour. Before data collection, the participants walked on the treadmill for 3 min to familiarize themselves with treadmill walking. Treadmill walking speed was reduced if the participant felt that the speed was not a comfortable walking speed.

At least three trials were conducted with two to five strides in each trial. Of the captured trials, the trials at comfortable walking speeds were averaged and analyzed. A minimum of 5 strides were averaged and used for analysis (7, 8).

To characterize gait patterns, the GGI and its 16 distinct gait parameters were used, according to the procedure described by Schutte (18) GGI is a single number, derived from gait kinematics and spatio-temporal parameters that quantify the deviation of pathological gait from normal gait. The GGI was calculated separately for right (RL) and left (LL) lower limbs based on the 16 gait parameters taken from the objective three-dimensional gait analysis (3DGA), including: (1) stance phase expressed as a percentage of gait cycle; (2) walking speed normalized to leg length; (3) cadence; (4) mean pelvic tilt; (5) range of motion (ROM) of pelvic tilt; (6) mean pelvic rotation; (7) minimum hip flexion; (8) ROM of hip flexion/extension; (9) peak hip abduction in swing; (10) mean hip rotation in stance; (11) knee flexion at initial contact; (12) time to peak knee flexion in swing expressed as the percentage of the gait cycle; (13) ROM of knee flexion; (14) peak dorsiflexion in stance; (15) peak dorsiflexion in swing; and (16) mean foot progression (18).

### Statistical Analysis

Normality of the analyzed parameters distribution was assessed using skewness and kurtosis and the Shapiro–Wilk test. Descriptive statistics were calculated for the clinical characteristics of both experimental and reference groups. Normally distributed variables are reported as means and standard deviations, and non-normally distributed variables are presented as the medians and ranges.

Non-hierarchical *k*-means clustering was used in the selection of the GGI for RL, LL, and bilateral lower limbs (BL) and the 16 distinct GGI gait parameters, assuming three clusters (22). K-means clustering is used with previously formed hypotheses concerning the number of clusters in the cases or variables. Because gait pattern was assumed to depend on 3 postural patterns recognized in our previous study (22), the identification of 3 clusters in the cluster analysis was justified.

The mean and standard deviation (SD) values for GGI RL, LL, BL, and the 16 GGI gait parameters were calculated for the entire group and for each of the 3 clusters and were compared among the subgroups. Analysis of variance (ANOVA, Tukey's *post-hoc* test) was used to detect differences in the aforementioned parameters. Only significant differences (*P* < 0.05) among the clusters were reported. In order to compute achieved statistical power in presented research the power analysis via GPower 3.1.9.2 was conducted. The computation was based on fixed effects, one-way ANOVA, which was later used to analyze group differences in GGI and 16 chosen parameters. For the power analysis, the mean effect size—partial eta-squared—across all conducted ANOVAs was calculated (mean of η_p_ = 0.40), α error probability was set to 0.05, total sample size was equal to 58 and the number of groups to 4. Output showed following values of parameters: Critical *F*_(3, 54)_ = 2.78 and achieved power (1 – β) was equal to 0.9997.

Power analysis was also calculated for the lowest significant effect size (η_p_ = 0.08) and the highest observed effect size (η_p_ = 0.91). Input parameters as α error probability, total sample size and number of groups were kept the same as in previous calculation. Achieved power (1 – β) in case of the lowest effect size was equal to 0.42 and for the highest observed effect sized power was equal to 1.00.

Based on the results of our previous study (9), the same group of participants were divided into four subgroups according to their postural patterns: (1) one subgroup of children with neutral posture (NP) represented by CTD (children typically developing; 45, 44%). Three subgroups of children with BCP were created as follows:

1. children with lordotic postural patterns, corresponding to forward-leaning posture (AP) (26; 25%)
2. children with swayback postural patterns, corresponding to backward-leaning posture (PP) (17; 16%)
3. children with balanced postural patterns, corresponding to balanced posture (BP) (15; 15%).

## Results

### Results of the GGI

In accordance with the GGI scores cluster analysis results, 30 (29%) participants were classified into Cluster 1; 23 (22%) were included in Cluster 2, 45 (44%) were classified in Cluster 3, whereas only 5 (5%) were included in Cluster 4 (Table 1). There were significant differences in the means of the various clusters for both singular GGI parameters and total GGI, as shown in Table 2.

**Table 1:** Gillette Gait Index (GGI).

**Subgroup**	**Cluster 1**	**Cluster 2**	**Cluster 3**	**Cluster 4**	**Total**
	***n* (%)**	***n* (%)**	***n* (%)**	***n* (%)**	***n* (%)**
NP	0 (0)	0 (0)	45 (100)	0 (0)	45 (44)
PP	15 (94)	0 (0)	0 (0)	2 (6)	17 (16.5)
BP	13 (81)	0 (0)	0 (0)	3 (19)	16 (13.5)
AP	2 (8)	23 (92)	0 (0)	0 (0)	25 (26)
Total	30 (29)	23 (22)	45 (44)	5 (5)	103 (100)

*Non-hierarchical k-means clustering*.

*Children with bilateral cerebral palsy (BCP) were classified according to their spinal profiles as follows: PP, swayback postural pattern; BP, balanced postural pattern; AP, lordotic postural pattern. Typically developing children were classified as having a neutral posture (NP)*.

**Table 2:** ANOVA results and differences between the means of the various GGI clusters of the left, right, and bilateral lower limbs.

**GGI**	**Groups**	**Sum of squares**	**df**	***F***	***P***
Lower Limbs	Between	96.26266	3	838.9144	0.00000
	Within	5.737335	100	–	–
	Total	60219.9	102	–	–
Left LL	Between	92.94822	3	513.425	0.00000
	Within	9.051781	100	–	–
	Total	14142.89	102	–	–
Right LL	Between	87.31906	3	297.3892	0.00000
	Within	14.68094	100	–	–
	Total	14142.89	102	–	–

*ANOVA, analysis of variance; GGI, Gillette Gait Index; LL, lower limbs*.

Tukey's *post-hoc* test revealed that the GGIs calculated for both LL and total GGI

reliably differentiated Cluster 1 from Clusters 2, 3, and 4; Cluster 2 from Cluster 3 and 4; and Cluster 3 from Cluster 4 via the cluster means (each *P* < 0.001). Significantly low values of GGI for BL and total GGI compared to all others were noted in the subjects included in Cluster 3 (Table 3).

**Table 3:** GGI scores representing the mean values for the lower limbs in children with bilateral cerebral palsy in specified clusters.

**Cluster**	***N***	**Mean**	**Min**	**Max**
1	30	184.21 ± 52.43	77.00	280.70
2	23	234.09 ± 10.35	148.5	381.33
3	45	12.34 ± 3.44	5.18	21.69
4	5	161.77 ± 40.20	76.99	235.17
Total	103	118.23 ± 103.34	5.18	381.33

*GGI, Gillette Gait Index*.

Cluster 3 was predominantly characterized by the NP subgroups consisting of the CTD group. The other three Clusters included only children with BCP. Cluster 1 was characterized by two subgroups of children with PP and BP, whereas Cluster 4 comprised only 4 participants from all BCP subgroups (Table 1).

Two gait patterns, by degree of deviation from normal gait, were recognized in the GGI calculation. One cluster (Cluster 3, *n* = 45) contained only CTD children (100%) and the second included children with BCP and gait patterns with lordotic postural patterns (92%). The gait patterns of children with PP and BP were not recognized using the cluster analysis.

### Results of the 16 Distinct GGI Gait Parameters

Using a factorial analysis, 7 variables from the 16 distinct GGI gait parameters were extracted including: (1) cadence, (2) mean pelvic tilt; (3) mean pelvic rotation, (4) minimum left and right hip flexion, (5) peak left and right hip abduction in swing; (6) left and right knee flexion at initial contact, and (7) peak dorsiflexion of left and right leg in stance.

In the cluster analysis results, 47 (46%) participants were classified into Cluster 1, 15 (14.5%) in Cluster 2, 16 (15.5 %) in Cluster 3, and 25 (24%) in Cluster 4 (Table 4). Four gait patterns emerged in accordance with postural patterns NP, PP, BP, and AP and were found to clearly correspond to the cluster patterns defined as follows:

1. normal gait (NG) corresponded to neutral posture (NP)
2. balanced gait pattern (BG) corresponded to balanced posture (BP)
3. lordotic gait pattern (AG) corresponded to lordotic posture (AP)
4. swayback gait pattern (PG) corresponded to backward-leaning posture (PP)

**Table 4:** Gait parameters in the GGI.

**Subgroup**	**Cluster 1**	**Cluster 2**	**Cluster 3**	**Cluster 4**	**Total**
	***n* (%)**	***n* (%)**	***n* (%)**	***n* (%)**	***n* (%)**
NP	45 (100)	0 (0)	0 (0)	0 (0)	45 (44)
PP	0 (0)	15 (88)	2 (12)	0 (0)	17 (16)
BP	1 (7)	0 (0)	14 (93)	0 (0)	15 (15)
AP	1 (4)	0 (0)	0 (0)	25 (96)	26 (25)
Total	47 (46)	15 (14.5)	16 (15.5)	25 (24)	103 (100)

*Non-hierarchical k-means clustering*.

*Children with bilateral cerebral palsy (BCP) were classified according to their spinal profiles as follows: PP, swayback postural pattern; BP, balanced postural pattern; AP, lordotic postural pattern. Typically developing children were classified as having a neutral posture (NP)*.

*GGI, Gillette Gait Index*.

There were significant differences among the means of the various clusters for all kinematics, as shown in Table 5. Table 5 shows the F values and significance levels; all differences between the means were significant.

**Table 5:** Results of the analysis of variance.

**Parameter**	**Groups**				**Statistical analysis**			
	**C1**	**C2**	**C3**	**C4**	**Sum of squares**	**df**	***F***	***post-hoc***
Cad (step/s)	1.24 ± 0.16	0.71 ± 0.23	0.76 ± 0.09	0.81 ± 0.13	29.06	3	19.92	C1-C2;C1-C3;C1-C4;C2-C4;C3-C4
MPT (°)	7.05 ± 4.19	5.36 ± 2.86	1.12 ± 6.26	−6.29 ± 5.52	64.01	3	84.24	C1-C2;C1-C3;C1-C4;C2-C4;C3-C4
MPR (°)	11.3 ± 6.32	5.57 ± 2.25	1.10 ± 6.67	−0.46 ± 3.23	54.98	3	58.46	C1-C2;C1-C4;C2-C3;C2-C4;C3-C4
MHF (°)	−11.3 ± 6.32	5.36 ± 2.86	−6.80 ± 10.79	−5.03 ± 3.57	20.91	3	12.89	C1-C2;C1-C3;C1-C4;C2-C4;C3-C4
PHAS (°)	2.24 ± 2.58	10.30 ± 5.61	3.24 ± 3.58	−8.75 ± 6.06	72.12	3	85.57	C1-C2;C1-C3;C1-C4;C2-C4;C3-C4
KFIC (°)	5.44 ± 4.81	22.08 ± 11.68	3.24 ± 3.58	17.94 ± 7.68	54.06	3	56.37	C1-C2;C1-C3;C1-C4;C2-C4;C3-C4
PDFS (°)	12.02 ± 3.54	16.90 ± 3.68	17.73 ± 3.68	−14.01 ± 8.69	87.39	3	299.14	C1-C2;C1-C3;C1-C4;C2-C4;C3-C4

*Differences between the means of various clusters with respect to kinematics (joint angles) are shown. Values are presented as means for each group (Cluster 1—C1, Cluster 2—C2, Cluster 3—C3, and Cluster 4—C4). Data are presented together with group interactions (estimated in a post-hoc analysis)*.

*Cad (step/s), cadence (step/s); MPT (°), mean pelvic tilt; MPR (°), mean pelvic rotation; MHF (°), minimum hip flexion; PHAS (°), peak hip abduction in swing; KFIC (°), knee flexion at initial contact; PDFS (°), peak dorsiflexion in stance*.

Cluster 1 contained solely CTD (100%). Cluster 4 almost entirely included children with AP (96%), whereas Cluster 2 included gait patterns of children with PP (88%), and cluster 3 included children with BP (93%) (Table 4).

Tukey's *post-hoc* test revealed that seven grouping variables from 16 distinct GGI gait parameters reliably differentiated all groups: Cluster 1 was distinct from Cluster 2, Cluster 3, and Cluster 4 which were also all distinct from each other based on cluster means (*P* = 0.00000 for each comparison) (Table 5).

## Discussion

The mean index for the TDC group was the lowest, and oscillated from 5.2 to 21.7. The mean index for all children with BCP ranged between 77.00 and 381.33. Although our range of GGI values of children with BCP was definitely lower, as reported by Schutte and Gage (18, 19), it is due to selection criteria involving only GMFCS I and II patients. Mean GGI values were significantly different between TDC and all subgroups of CP.

In this study, the cluster analysis differentiated between the gait of the TDC and BCP groups, and the pathological gait patterns in the BCP group. Our findings show that three clusters differentiated pathological from normal gaits in children with BCP, and corresponded with a postural category. However, only the forward-leaning posture (AP) was clearly separated (cluster 2). No overlap was found between GGI values for AP children and all other individuals with BCP. In contrast, GGI values for BP and PP postural categories overlapped within one cluster (cluster 1).

The *post-hoc* analysis showed that children with AP presented with significantly higher degrees of deviation from normal gait than other children with BCP (Table 2). We expected that GGI could distinguish between patients with BCP in all postural categories due to the wide range of GGI values in BP and PP; however, no statistical conclusions could be made about the relationship between those values and GGI values.

Estimating the differences between gait patterns corresponding to postural patterns in BCP is promising. Thus, evaluating GGI gait biomechanics was next step. The cluster analysis revealed 4 gait patterns, defined by non-overlapping kinematics associated with four postural categories: (1) normal gait corresponding to TDC neutral postures (NG); and 3 gait patterns in BCP corresponded to these postural patterns: (1) balanced gait pattern corresponded to balanced posture (BG); (2) lordotic gait pattern corresponded to forward-leaning posture (AG); and (3) swayback gait pattern corresponded to backward-leaning posture (PG). There were significant differences among the gait patterns for all 8 GGI gait parameters including: cadence, mean pelvic tilt; mean pelvic rotation, minimum hip flexion, peak hip abduction in swing; knee flexion at initial contact, and peak dorsiflexion in stance (Table 5). We found that discrepancies in BCP gait were not simply due to sagittal plane lower limb kinematic deviations (23–26). We also identified previously unreported kinematic deviations in BCP gait resulting from postural pattern features including: pelvic misalignment in sagittal and horizontal planes and an inadequate swing phase in hip abduction. This distinguished between three gait patterns in children with BCP. In AG, PG, and BG, inadequate mean pelvic tilt (deficits in AG and BG; and excess in PG) during the gait cycle was observed. Static misalignment of the pelvis in the sagittal plane, such as with excessive anterior pelvic tilt and excessive lumbar curves in the spines of children with forward-leaning postures; extreme hyperlordosis and postural deviations in the horizontal planes, characteristic of children with balanced postures; and excessive posterior pelvic tilt and insufficient lumbar curves in the spines in children with backward-leaning postures may result in pelvic tilt deficits and pelvic motion deviations during the gait cycle in these children (9). These observations are consistent with gait cycle pelvic motion deficits in the horizontal plane, as revealed in all three pathological gait patterns. The static sagittal misorientations of the pelvis, such as with excessive anterior (AG), insufficient posterior (BG), and excessive posterior (PG) pelvic tilts are perhaps the reasons for pelvic rotation deficits in all pathological gait patterns. Only the netural pelvis position in the sagittal plane promotes proper pelvic rotation while walking (19).

As expected, differences in lower limb sagittal plane kinematics between the gait patterns were observed. While kinematics in TDC oscillated within the normal range, 3 pathological sets of lower limb kinematic values at the hip (minimum hip flexion and peak hip abduction in swing), knee (knee flexion at initial contact), and ankle (peak dorsiflexion on stance) were noted in children with BCP. Two gait patterns, AG and PG, opposed each other due to hip abductions in swing and dorsiflexion. Stance deviations from normal gait drew special attention. These differences can be summarized as insufficient hip abduction in swing and insufficient dorsiflexion in stance for AG, and excessive hip abduction in swing and excessive dorsiflexion in stance for PG.

To the best of our knowledge, this study is the first to recognize gait patterns corresponding to postural pattern in children with BCP. The classification of children with BCP into four subgroups according to their postural patterns was critically important in our analysis. Although several studies have reported gait pattern classifications based on lower limb kinematic deviations in children with BCP, they do not account for their pelvic and truncal postures. This makes it difficult to compare the results obtained herein, with those found in previous studies on gait analysis.

Typical overground walking speed (spontaneous) of and time taken to walk 10 m by each subject were collected before the gait analysis. Based on the spontaneous speed of walking, treadmill belt speeds were calculated as values in kilometers per hour. Before data collection, the participants walked on the treadmill for 3 min to familiarize themselves with treadmill walking. Treadmill walking speed was reduced if the participant felt that the speed was not a comfortable walking speed.

## Study limitations

A limitation of the present study is that the ability to walk without assistive devices was a criterion for study inclusion. Additionally, previous lower limb surgery was an exclusion criterion, which is why only some of the children with BCP were included. Another limitation can be that this study was based on treadmill-based gait analysis. However, the findings from treadmill-based gait research can differ from overground gait data in children with CP. Despite, that many studies have investigated the differences between the treadmill and overground walking in children with CP, conclusive research has not been conducted to verify this assumption. Although, it is generally agreed that the treadmill walking can alter spatiotemporal gait variables, there still exist inconsistencies as to the kinematic differences between the two walking modalities.

## Conclusion

Despite the above-mentioned limitations, our findings clearly suggest that children with BCP exhibit different gait patterns corresponding to their postural patterns. Moreover, we described basic differences in the BCP gait that corresponded with different postural patterns. This information may help improve the guidelines for establishing therapy in children with BCP before the pathological gait is fully established. These findings should provide an essential step toward recognizing further kinematic differences between gaits in a follow-up study.

## Data Availability

All datasets generated for this study are included in the manuscript and/or the supplementary files.

## Ethics Statement

This study was carried out in accordance with the recommendations of Bioethics Committee of Medical University of Silesia with written informed consent from all subjects. All subjects gave written informed consent in accordance with the Declaration of Helsinki. The protocol was approved by the Bioethics Committee of Medical University of Silesia.

## Author Contributions

MD-S and AS contributed conception and design of the study. AS organized the database. MD-S performed the statistical analysis and wrote the first draft of the manuscript. MD-S and AS wrote sections of the manuscript. All authors contributed to manuscript revision, read, and approved the submitted version.

### Conflict of Interest Statement

The authors declare that the research was conducted in the absence of any commercial or financial relationships that could be construed as a potential conflict of interest.

## References

[1] Bax M Goldstein M Rosenbaum P Leviton A Paneth N Dan B Executive committee for the definition of cerebral palsy. proposed definition and classification of cerebral palsy, April 2005. Dev Med Child Neurol. (2005) 47:571–6. 10.1017/S001216220500112X 16108461

[2] Rosenbaum P Paneth N Leviton A Goldstein M Bax M Damiano D. A report: the definition and classification of cerebral palsy April 2006. Dev Med Child Neurol. (2007) 109:8–14. 10.1111/j.1469-8749.2007.00001.x 17370477

[3] Surveillance of Cerebral Palsy in Europe (SCPE) Surveillance of cerebral palsy in Europe: a collaboration of cerebral palsy surveys and registers. Dev Med Child Neurol. (2000) 42:816–24. 10.1111/j.1469-8749.2000.tb00695.x 11132255

[4] Domagalska–Szopa M Szopa A. Postural pattern recognition in children with unilateral cerebral palsy. Ther Clin Risk Manag. (2014) 10:113–20. 10.2147/TCRM.S58186 24600228 PMC3933466

[5] Domagalska-Szopa M Szopa A. Body posture asymmetry differences between children with mild scoliosis and children with unilateral cerebral palsy. Biomed Res Int. (2013) 2013:462094. 10.1155/2013/462094 24224163 PMC3810063

[6] Domagalska M Szopa A Lembert D. A descriptive analysis of abnormal postural patterns in children with hemiplegic cerebral palsy. Med Sci Monit. (2011) 17:110–6. 10.12659/MSM.881396 21278687 PMC3524706

[7] Szopa A Domagalska–Szopa M Czamara A. Gait pattern differences in children with unilateral cerebral palsy. Res Dev Disabil. (2014) 35:2261–6. 10.1016/j.ridd.2014.05.020 24946266

[8] Domagalska-Szopa M Szopa A. Gait pattern differences between children with mild scoliosis and children with unilateral cerebral palsy. PLoS ONE. (2014) 9:e103095. 10.1371/journal.pone.0103095 25089908 PMC4121082

[9] Domagalska-Szopa M Szopa A. Postural orientation and standing postural alignment in ambulant children with bilateral cerebral palsy. Clin Biomech. (2017) 49:22–7. 10.1016/j.clinbiomech.2017.08.005 28830044

[10] Sutherland DH Davids JR Common gait abnormalities of the knee in cerebral palsy. Clin Orthop Relat Res. (1993) 288:139–47. 8458127

[11] Dobson F Morris ME Baker R Graham HK. Gait classification in children with cerebral palsy: a systematic review. Gait Posture. (2007) 25:140–52. 10.1016/j.gaitpost.2006.01.003 16490354

[12] Ounpuu S Deluca P Davis RB Gait analysis. In: Neville B Goodman R editors. Congenital Hemiplegia (Clinics in Developmental Medicine). London: MacKeith Press (2000). p. 81–97.

[13] Rodda J Graham HK. Classification of gait patterns in spastic hemiplegia and spastic diplegia: a basis for a management algorithm. Eur J Neurol. (2001) 8:98–108. 10.1046/j.1468-1331.2001.00042.x 11851738

[14] Rodda JM Graham HK Carson L Galea MP Wolfe R. Sagittal gait patterns in spastic diplegia. J Bone Joint Surg Br. (2004) 86:251–8. 10.1302/0301-620X.86B2.13878 15046442

[15] Toro B Nester CJ Farren PC. Cluster analysis for the extraction of sagittal gait patterns in children with cerebral palsy. Gait Posture. (2007) 25:157–65. 10.1016/j.gaitpost.2006.02.004 16647260

[16] Bonnefoy-Mazure A Sagawa Y. Jr Lascombes P De Coulon G Armand S. Identification of gait patterns in individuals with cerebral palsy using multiple correspondence analysis. Res Dev Disabil. (2013) 34:2684–93. 10.1016/j.ridd.2013.05.002 23770664

[17] Sangeux M Rodda J Graham HK Sagittal gait patterns in cerebral palsy: the plantar exor-knee extension couple index. Gait Posture. (2015) 41:586–91. 10.1016/j.gaitpost.2014.12.019 25604121

[18] Schutte LM Narayanan U Stout JL Selber P Gage JR Schwartz MH. An index for quantifying deviations from normal gait. Gait Posture. (2000) 11:25–31. 10.1016/S0966-6362(99)00047-8 10664482

[19] Gage JR. The Treatment of Gait Problems in Cerebral Palsy. London: MacKeith Press (2004). 11727367

[20] Romei M Galli M Motta F Schwartz M Crivellini M. Use of the normalcy index for the evaluation of gait pathology. Gait Posture. (2004) 19:85–90. 10.1016/S0966-6362(03)00017-1 14741307

[21] Schwartz M The reliability of the normalcy index for quantifying gait pathology. Gait Posture. (2003) 18:S119. 10.1016/S0966-6362(03)00073-0 14741307

[22] Davidson I Understanding K-Means Non-hierarchical Clustering. Albany, NY: SUNY (2002).

[23] Lin CJ Guo LY Su FC Chou YL Cherng RJ. Common abnormal kinetic patterns of the knee in gait in spastic diplegia of cerebral palsy. Gait Posture. (2000) 11:224–32. 10.1016/S0966-6362(00)00049-7 10802435

[24] Rozumalski A Schwartz MH. Crouch gait patterns defined using k-means cluster analysis are related to underlying clinical pathology. Gait Posture. (2009) 30:155–60. 10.1016/j.gaitpost.2009.05.010 19535249

[25] Becher J Pediatric rehabilitation in children with cerebral palsy: general management, classification of motor disorders. J Prosthet Orthot. (2002) 14:143–9. 10.1097/00008526-200212000-00004

[26] Assi A Ghanem I Lavaste F Skalli W Gait analysis in children and uncertainty assessment for Davis protocol and Gillette Gait Index. Gait Posture. (2009) 3:22–6. 10.1016/j.gaitpost.2009.02.011 19321345

